# Activation of TAS2R Signaling by Diphenidol Suppresses Tumor Growth and Remodels the Tumor Immune Microenvironment in Oral Squamous Cell Carcinoma

**DOI:** 10.3390/cancers18101527

**Published:** 2026-05-09

**Authors:** Nisrina Ekayani Nasrun, Akihiko Tanimura, Koki Yoshida, Osamu Uehara, Yuki Kunisada, Kiyofumi Takabatake, Akihiro Hosoya, Hiroaki Takebe, Hitoshi Nagatsuka, Yoshihiro Abiko, Muhammad Ruslin, Tsuyoshi Shimo

**Affiliations:** 1 Division of Reconstructive Surgery for Oral and Maxillofacial Region, Department of Human Biology and Pathophysiology, School of Dentistry, Health Sciences University of Hokkaido, 1757 Kanazawa, Tobetsu 061-0293, Japan; nisrina@unhas.ac.id; 2 Department of Oral and Maxillofacial Surgery, Faculty of Dentistry, Hasanuddin University, Makassar 90245, Indonesia; mruslin@unhas.ac.id; 3 Division of Pharmacology, Department of Oral Biology, School of Dentistry, Health Sciences University of Hokkaido, Tobetsu 061-0293, Japan; tanimura@hoku-iryo-u.ac.jp; 4 Division of Oral Medicine and Pathology, Department of Human Biology and Pathophysiology, School of Dentistry, Health Sciences University of Hokkaido, 1757 Kanazawa, Tobetsu 061-0293, Japan; denty@hoku-iryo-u.ac.jp (K.Y.); yoshi-ab@hoku-iryo-u.ac.jp (Y.A.); 5 Division of Disease Control and Molecular Epidemiology, Department of Oral Growth and Development, School of Dentistry, Health Sciences University of Hokkaido, 1757 Kanazawa, Tobetsu 061-0293, Japan; osamu@hoku-iryo-u.ac.jp; 6 Department of Oral and Maxillofacial Surgery, Okayama University Graduate School of Medicine, Dentistry and Pharmaceutical Sciences, Okayama 700-8525, Japan; de16013@s.okadai.jp; 7 Department of Oral Pathology and Medicine, Okayama University Graduate School of Medicine, Dentistry and Pharmaceutical Sciences, Okayama 700-8525, Japan; gmd422094@s.okayama-u.ac.jp (K.T.); jin@okayama-u.ac.jp (H.N.); 8 Division of Craniofacial Development and Tissue Biology, Tohoku University Graduate School of Dentistry, Sendai 980-8575, Japan; akihiro.hosoya.a6@tohoku.ac.jp; 9 Division of Histology, Department of Oral Biology, School of Dentistry, Health Sciences University of Hokkaido, 1757 Kanazawa, Tobetsu 061-0293, Japan; takebeh@hoku-iryo-u.ac.jp

**Keywords:** bitter taste receptor, diphenidol, immunometabolism, oral squamous cellcarcinoma, TAS2R signaling, tumor immune microenvironment

## Abstract

**Background**: Oral squamous cell carcinoma (OSCC) remains a clinically challenging malignancy characterized by aggressive behavior and limited therapeutic options. Bitter taste receptors (TAS2Rs), expressed across multiple tissues and cancer types, have recently emerged as regulators of tumor biology and immune responses; however, their functional significance in OSCC remains poorly understood. **Methods**: Immunohistochemical analysis was performed using surgically resected human tongue OSCC specimens and a tissue microarray (TMA) cohort. In parallel, four TAS2R agonists were evaluated in SCC7 cells to assess intracellular calcium responses. RNA sequencing was conducted to analyze transcriptional changes following diphenidol treatment, and functional assays, including proliferation, migration, and apoptosis analyses, were performed in vitro. Antitumor effects were further evaluated in a syngeneic SCC7 mouse model, followed by TUNEL staining and flow cytometry to assess apoptosis and immune cell infiltration. **Results**: TAS2R38 expression was markedly upregulated in dysplastic and invasive OSCC lesions with predominant nuclear localization and was associated with histological grade and clinical stage, indicating an early and sustained alteration during tumor progression. Among the agonists tested, diphenidol most strongly induced IP_3_-dependent intracellular Ca^2+^ elevation. RNA sequencing revealed upregulation of Il1rl1 and Lzts2. Functionally, diphenidol significantly suppressed SCC7 cell proliferation and migration and induced apoptosis in vitro. In vivo, diphenidol reduced tumor volume and weight and increased apoptotic activity. Flow cytometry demonstrated a marked reduction in tumor-infiltrating CD4^+^CD25^+^Foxp3^+^ regulatory T cells, indicating modulation of the tumor immune microenvironment. **Conclusions**: TAS2R activation by diphenidol suppresses tumor growth through both tumor-intrinsic mechanisms and modulation of the tumor immune microenvironment in OSCC. These findings define TAS2R-mediated calcium signaling as a novel axis linking tumor progression and immunoregulation. Given that diphenidol is a clinically approved drug with an established safety profile, our results provide a strong rationale for TAS2R-targeted drug repurposing strategies in cancer therapy.

## Simple Summary

Oral squamous cell carcinoma (OSCC) remains a difficult-to-treat cancer, highlighting the need for new therapeutic strategies. Bitter taste receptors (TAS2Rs), which are expressed in various tissues and cancers, have recently been implicated in the regulation of tumor behavior and the tumor immune microenvironment. In this study, we evaluated multiple TAS2R agonists and identified diphenidol as the most effective compound for inducing intracellular Ca^2+^ signaling in OSCC cells. Diphenidol inhibited tumor cell proliferation and migration and induced apoptosis in vitro. In a mouse model, it also increased apoptotic cell death and reduced regulatory T-cell infiltration within tumors, suggesting an improvement of the tumor immune microenvironment. These findings demonstrate that TAS2R activation can simultaneously regulate tumor growth and tumor immunity in OSCC, revealing a link between TAS2R-mediated calcium signaling and tumor immunometabolic regulation. Because diphenidol is already a clinically approved drug, our results highlight the potential of drug repurposing as a novel therapeutic strategy for OSCC.

## 1. Introduction

Oral squamous cell carcinoma (OSCC), a subtype of head and neck squamous cell carcinoma (HNSCC), constitutes over 90% of oral cavity malignancies and remains a major clinical challenge due to its aggressive behavior and limited therapeutic options. Despite advancements in multimodal therapies, including surgery, radiation, chemotherapy, and immune checkpoint inhibitors (ICIs), OSCC continues to exhibit a poor prognosis, with a five-year survival rate of approximately 50% in advanced cases and a high incidence of recurrence [1,2,3,4]. These outcomes are attributed to late diagnosis, tumor aggressiveness, and resistance to therapy. Consequently, the identification of novel molecular targets and the repurposing of approved agents with established safety profiles are imperative for the treatment of OSCC [4]. However, the molecular mechanisms underlying OSCC progression remain incompletely understood, particularly regarding mechanisms linking tumor progression to tumor–immune interactions.

The tumor microenvironment (TME) plays a critical role in tumor progression, immune evasion, and therapeutic resistance. It comprises a complex ecosystem of immune cells, fibroblasts, endothelial cells, and extracellular matrix components that collectively promote tumor growth and modulate immune responses [5,6,7,8]. Among the cellular components of the TME, regulatory T cells (Tregs) are known for their immunosuppressive function and are often enriched in OSCC tissues, correlating with poor prognosis and reduced efficacy of immunotherapy [9,10,11,12]. Accordingly, therapeutic strategies aimed at reprogramming the TME, particularly by mitigating Treg-mediated immunosuppression, are being actively investigated.

G protein-coupled receptors (GPCRs), the largest family of membrane receptors, are appealing pharmacological targets due to their druggability and involvement in cancer biology [13,14]. Bitter taste receptors, known as TAS2Rs in humans and Tas2rs in mice, form a subfamily of GPCRs initially discovered in gustatory tissues. However, they are now recognized for their expression in non-sensory tissues, such as airways, immune cells, and malignancies [15]. Activation of TAS2Rs has been linked to the modulation of intracellular calcium (Ca^2+^) signaling, inflammatory responses, and apoptosis [16,17].

Among the members of this receptor family, TAS2R38 is one of the most extensively studied subtypes and has been implicated in immune modulation and epithelial barrier function; however, its role in the pathogenesis of oral squamous cell carcinoma (OSCC) remains largely unexplored. TAS2R38 has been widely investigated in terms of genetic polymorphisms and functional variability, including well-characterized haplotypes (e.g., PAV and AVI), and has been associated with disease susceptibility and immune regulation across various tissues [15,16,17]. In addition, preliminary observations and public database analyses suggest that TAS2R38 expression may be associated with tumor progression and immune-related features in head and neck cancers. Based on these considerations, we focused on TAS2R38 as a representative TAS2R subtype in this study, as it was also identified in our preliminary analyses as one of the TAS2R subtypes showing notable expression changes, further supporting its selection for functional investigation in OSCC.

Our previous work demonstrated that sonic hedgehog (SHH) secreted by OSCC cells activates hedgehog signaling in osteoclasts and promotes jawbone destruction [18]. Interestingly, pathway analysis of hedgehog-stimulated osteoclasts revealed the upregulation of TAS2R-related genes. These findings raised the possibility that TAS2R signaling may interact with hedgehog-associated pathways within the OSCC microenvironment.

Diphenidol is a clinically approved antiemetic agent that functions as a non-selective TAS2R agonist. It has been shown to trigger intracellular Ca^2+^ responses in non-gustatory cells [19,20]. TAS2R agonists may be implicated in the modulation of multiple oncogenic pathways, including those involving PI3K/AKT, MAPK, and hedgehog [21,22,23]. However, the role of TAS2R signaling in OSCC progression and its potential impact on the tumor immune microenvironment remain poorly understood.

In this study, we aimed to determine whether the bitter taste receptor agonist diphenidol exerts antitumor effects in OSCC via calcium-dependent signaling and immune modulation. To elucidate the underlying mechanisms, we performed calcium imaging and RNA-seq analyses to characterize the transcriptomic alterations in diphenidol-treated OSCC cells. The antiproliferative, pro-apoptotic, and anti-migratory effects of diphenidol were evaluated in vitro, whereas its therapeutic efficacy and impact on regulatory T cell infiltration were examined in vivo using a syngeneic SCC7 mouse model. Our findings provide new insights into TAS2R-associated antitumor mechanisms and support the therapeutic potential of diphenidol as a repositioned agent for OSCC treatment.

## 2. Materials and Methods

### 2.1. Patient Samples and Ethical Approval

**Figure 1:**
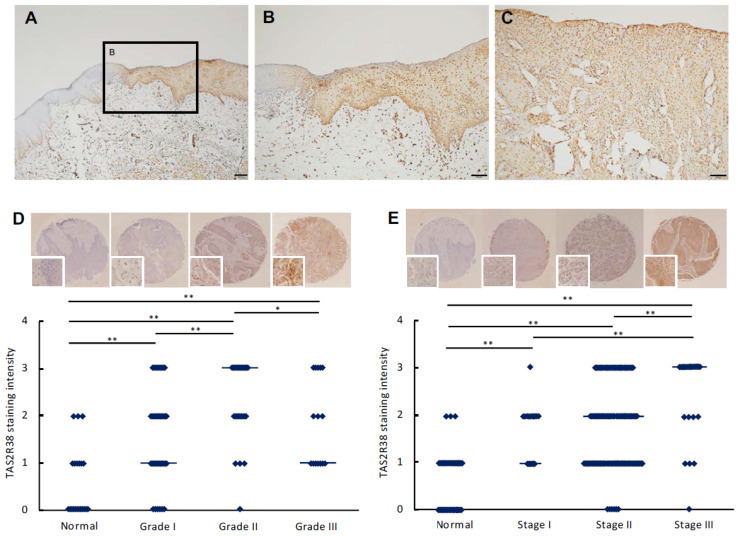
TAS2R38 expression in human tongue oral squamous cell carcinoma (OSCC). (**A**–**C**) Representative immunohistochemical staining of TAS2R38 in surgically resected human tongue OSCC specimens (*n* = 10). (**A**) Histologically normal tongue epithelium located at the surgical margin showing weak TAS2R38 expression. (**B**) Increased TAS2R38 immunoreactivity in dysplastic lesions. (**C**) Progressive upregulation and prominent nuclear localization of TAS2R38 in invasive tumor cells. Representative images were selected from typical staining patterns observed across the analyzed cases. Scale bars: (**A**) 200 μm; (**B**) 100 μm; (**C**) 50 μm. (**D**) Semiquantitative analysis of TAS2R38 staining intensity according to histological grade in tongue OSCC TMA. Upper panels show representative TMA cores stained for TAS2R38, with corresponding higher-magnification images indicated by boxed regions. Cases included normal tongue epithelium (*n* = 27), Grade 1 (*n* = 51), Grade 2 (*n* = 24), and Grade 3 (*n* = 9). Quantitative analysis was performed using pooled evaluable TMA spots (*n* = 222), as described in the Methods section. Each dot represents an individual TMA spot, and horizontal lines indicate the median. (**E**) Semiquantitative analysis of TAS2R38 staining intensity according to clinical stage in tongue OSCC TMA. Upper panels show representative TMA cores with corresponding magnified images (boxed regions). Cases included normal tongue epithelium (*n* = 27), Stage I (*n* = 9), Stage II (*n* = 63), and Stage III (*n* = 12). Quantitative analysis was performed using pooled evaluable TMA spots (*n* = 222), as described in the Methods section. Each dot represents an individual TMA spot, and horizontal lines indicate the median. For the present analysis, only tongue OSCC cases were selected from the TMA cohort. Staining intensity was scored on a four-point scale (0 = weak, 1 = moderate, 2 = strong, 3 = very strong). Statistical analysis was performed using one-way ANOVA followed by Tukey’s multiple comparison test (* *p* < 0.05, ** *p* < 0.01).

Formalin-fixed, paraffin-embedded tissue samples of OSCC were obtained from the Department of Oral Pathology, Okayama University. In this study, ten surgically resected primary tongue OSCC specimens were analyzed by immunohistochemistry to evaluate TAS2R38 expression patterns, and representative images from these cases are presented in Figure 1. Immunohistochemical staining was independently evaluated by experienced oral pathologists. This study was approved by the Ethics Committee of the Okayama University Graduate School of Medicine, Dentistry and Pharmaceutical Sciences (approval code: 1703-042-001) and conducted in accordance with the Declaration of Helsinki. All patients provided written informed consent. None of the patients received preoperative chemotherapy, radiotherapy, or immunotherapy.

### 2.2. Tissue Microarray Analysis

A commercially available human OSCC tissue microarray (TMA) (OR208a; U.S. Biomax, Derwood, MD, USA) was analyzed by immunohistochemistry. Only tongue squamous cell carcinoma (tongue OSCC) cases and normal tongue tissue were selected from the TMA for analysis, and OSCCs from other anatomical sites were excluded. Formalin-fixed, paraffin-embedded (FFPE) sections (3 μm) were deparaffinized in xylene, rehydrated in ethanol, and subjected to heat-mediated antigen retrieval in citrate buffer (pH 6.0). Endogenous peroxidase activity was quenched with 0.3% H_2_O_2_, followed by blocking and overnight incubation at 4 °C with anti-TAS2R38 antibody (Thermo Fisher Scientific, Waltham, MA, USA, cat #PA5-99928, 1:100). Immunoreactivity was visualized using the VECTASTAIN^®^ ABC Rabbit IgG Kit (Vector Laboratories, Newark, CA, USA; cat #PK-4001) and diaminobenzidine (DAB) (Nichirei Biosciences, Tokyo, Japan; cat #415192F). Staining intensity was evaluated visually by two blinded observers. For objective quantitative assessment, slides were scanned at 20× magnification and analyzed using Fiji/ImageJ software version 2.14.0/1.54m (National Institutes of Health [NIH], Bethesda, MD, USA). Tumor regions were manually selected by investigators blinded to clinical information. Staining intensity was graded on a four-level scale (0 = weak, 1 = moderate, 2 = strong, 3 = very strong). Pixel-based quantification of DAB-positive areas was performed, and the percentage of positive pixels for each intensity category was calculated after color deconvolution. The results obtained by visual evaluation and Fiji/ImageJ-based quantification were consistent. Representative images were acquired at 20× magnification. Quantitative analysis was performed using selected TMA regions (A1–18, B1–18, C1–18, G10–18, H1–18, I1–3, K1–18, L1–9), yielding 111 evaluable spots. The analysis was conducted in duplicate, and the combined dataset (*n* = 222 spots) was used for statistical evaluation. Staining intensity scores were treated as ordinal data; however, to enable multiple group comparisons across subgroups, parametric analysis (one-way ANOVA followed by Tukey’s post hoc test) was applied.

### 2.3. Reagents and Antibodies

The TAS2R agonists, amarogentin (Selleck Chemicals, Tokyo, Japan; cat #S1273), caffeine (Cayman Chemical, Ann Arbor, MI, USA; cat #14118), denatonium benzoate (Tokyo Chemical Industry, Tokyo, Japan; cat #D0176), and diphenidol (Tokyo Chemical Industry; cat #D1345) were dissolved in dimethyl sulfoxide (DMSO) (Sigma-Aldrich, St. Louis, MO, USA; cat #D2438) and stored at −20 °C. These TAS2R agonists were selected based on their reported activity against multiple TAS2R subtypes and their frequent use in functional TAS2R studies. Fura-2 acetoxymethyl ester (Fura-2AM) was purchased from Dojindo Laboratories (Kumamoto, Japan; cat #F015). ATP and thapsigargin were obtained from Fujifilm Wako Pure Chemical Co. (Osaka, Japan; cat #013-12061) and Sigma-Aldrich, respectively.

The following antibodies were purchased from BD Pharmingen™ (San Diego, CA, USA) for flow cytometric analysis: CD16/CD32 (cat #553141), CD3e (#570648), CD4 (#561091), CD8a (#564459), CD25 (#562606), Foxp3 (#560414), and PE-conjugated rat IgG2b isotype control (#553989). Tumor & Tissue Dissociation Reagent (TTDR; cat #661563) and Fixable Viability Stain 780 (FVS780; #565388) were also from BD Pharmingen™.

### 2.4. Cell Culture Conditions

SCC7, a mouse-derived squamous cell carcinoma cell line (RRID: CVCL_V412), was obtained from the Cell Bank of the Medical Cell Resource Center, Institute of Development, Aging and Cancer, Tohoku University. Cells were cultured in Dulbecco’s Modified Eagle Medium/Nutrient Mixture F-12 (DMEM/F12) (Sigma-Aldrich; cat #D6434) supplemented with 5% fetal bovine serum (FBS) (Biowest, Logan, UT, USA; cat #S1520), 10 μg/mL L-glutamine, and 10 μg/mL penicillin–streptomycin (Life Technologies, Carlsbad, CA, USA; cat #25030081 and #1514012). Cultures were maintained at 37 °C in a humidified incubator with 5% CO_2_. For in vitro experiments, only assays that met strict quality and reproducibility standards were included in the analysis. Cells were required to exhibit more than 90% viability and to be in the exponential growth phase at the time of diphenidol treatment. To minimize phenotypic drift, experiments were conducted using cell lines maintained at consistent passage numbers (≤20 passages from thawing). Each experimental condition was performed in triplicate to ensure reliability. Assays were excluded if mycoplasma contamination, abnormal cell morphology, or deviations in treatment concentration or duration were detected, or if replicate data deviated by more than two standard deviations from the group mean. All experiments were independently repeated at least three times to confirm reproducibility.

### 2.5. Measurement of Intracellular Ca^2+^

Intracellular calcium levels ([Ca^2+^]ᵢ) were measured using Fura-2-based ratiometric imaging. SCC7 cells were seeded in 60-mm dishes at 1 × 10^5^ cells/dish and cultured for 4–6 days. Cells were harvested and incubated for 30 min at room temperature with 2 μM Fura-2AM in HEPES-buffered HBSS (HBSS-H) containing 0.2% BSA. After washing, cells were resuspended in fresh HBSS-H and maintained at room temperature.

Fluorescence was measured at 37 °C using a Hitachi F-2500 spectrofluorometer (Hitachi, Tokyo, Japan) with excitation at 340/380 nm and emission at 510 nm. The [Ca^2+^]ᵢ was monitored following stimulation with TAS2R agonists or ATP. Maximum and minimum fluorescence ratios were obtained using 0.1% Triton X-100 and 5 mM EGTA, respectively.

### 2.6. RNA-Seq and Functional Enrichment Analysis

SCC7 cells were seeded at 4 × 10^5^ cells per 10-cm dish and treated with 0.1 mM diphenidol for 24 h. The concentrations used in subsequent experiments were selected based on preliminary dose–response analyses, the results of which are presented in the Results section. Total RNA was extracted using TRIzol reagent (Invitrogen, Carlsbad, CA, USA; cat #15596018CN) and purified using the RNeasy Mini Kit (Qiagen GmbH, Hilden, Germany; cat #74104). Samples were run on a BioAnalyzer to assess total RNA integrity. Only high-quality RNA samples (RNA integrity number ≥ 9.0) were used to construct the sequence library. PCR-based amplification was performed using the template prepared with the strand-specific library preparation method (dUTP method) and an index sequence-containing primer to prepare a sequence library (NEBNext Poly(A) mRNA Magnetic Isolation Module; NEBNext Ultra II Directional RNA Library Prep Kit for Illumina). RNA-seq data were obtained using the NovaSeq 6000 system (Illumina, San Diego, CA, USA). The sequence reads were trimmed using Trimmomatic (ver.0.38). Trimmed sequence reads were mapped to the reference genome (hg38) using HISAT2 (ver.2.1.0). The raw read count for each gene was calculated using featureCounts (ver.1.6.3). The raw read count was uploaded to iDEP.95 (http://bioinformatics.sdstate.edu/idep95/, accessed on 24 April 2024 and updated on 7 October 2025) for hierarchical clustering, principal component analysis (PCA), correlation evaluation, heat map creation, and functional enrichment analysis. Initial settings of iDEP were used for the analysis.

### 2.7. Quantitative Reverse Transcription PCR (qRT-PCR)

Complementary DNA (cDNA) was synthesized from total RNA using the ReverTra Ace^®^ qPCR RT Master Mix (Toyobo, Osaka, Japan; Cat# FSQ-201). Quantitative reverse transcription PCR (qRT-PCR) was performed on the LightCycler^®^ 96 system (Roche Diagnostics, Basel, Switzerland) using KAPA SYBR FAST qPCR Master Mix (Kapa Biosystems, Wilmington, MA, USA; Cat# SFUKB) and gene-specific primers (listed in Table S1). Thermal cycling conditions were as follows: initial denaturation at 95 °C for 3 min, followed by 40 cycles of denaturation at 95 °C for 10 s, annealing at 60 °C for 20 s, and extension at 72 °C for 1 s. Relative gene expression was calculated using the ΔΔCq method and normalized to β-actin.

### 2.8. Cell Proliferation and Cytotoxicity Assays

SCC7 cells were seeded at 4 × 10^3^ cells/well in 96-well plates and treated with diphenidol (0–3 mM) for 24, 48, or 72 h. The concentration range of diphenidol (0–3 mM) was selected based on preliminary dose–response experiments and previous studies evaluating TAS2R agonists in cultured cells, which reported that millimolar concentrations are required to elicit robust intracellular Ca^2+^ responses. Cell viability was assessed using the WST-1 assay (Thermo Fisher Scientific; cat #ab155902), with absorbance measured at 450 nm using a Multiskan FC microplate reader (Thermo Fisher Scientific, Waltham, MA, USA). Experiments were conducted in quadruplicate. To determine IC_50_ values, cells were harvested, stained with 0.4% trypan blue, and counted using a hemocytometer. Viable (unstained) and non-viable (blue) cells were quantified to assess diphenidol cytotoxicity.

### 2.9. Migration Assays

For migration analysis, SCC7 murine squamous carcinoma cells were detached with trypsin-EDTA, counted, and resuspended in complete growth medium. Cells were seeded into StenCell removable polydimethylsiloxane (PDMS) chambers (Nonet type; Idylle Labs, Paris, France), a silicone-based elastomeric system [24], affixed to 35-mm culture dishes. Approximately 2.0–4.0 × 10^5^ cells per well were plated, a density optimized to achieve a confluent monolayer surrounding the nonet posts within 12–24 h. Cells were cultured at 37 °C in a humidified atmosphere with 5% CO_2_ until reaching >95% confluence, as confirmed by phase-contrast microscopy. After 48 h, confluent monolayers were treated with diphenidol at concentrations ranging from 10^−6^ to 10^−3^ mM for 2 h. The StenCell PDMS insert was then carefully removed vertically using sterile forceps to generate a precisely defined, cell-free circular area without disturbing the surrounding monolayer. The culture medium was immediately replaced with pre-warmed, phenol-red-free DMEM (Sigma-Aldrich; cat #D4947) containing 1% FBS to suppress proliferation-driven closure and remove any detached cells. Subsequently, cell migration was monitored by time-lapse imaging. Cell imaging was performed using a Nikon ECLIPSE Ti2 inverted microscope (Nikon, Tokyo, Japan) equipped with a stage-top incubator maintained at 37 °C and 5% CO_2_. Images were acquired under brightfield illumination at defined intervals. After 48 h, endpoint images were captured to quantify cell migration. The migrated area was analyzed using NIS-Elements software version 5.21.00 (Nikon, Tokyo, Japan) and expressed as the difference between the area covered by cells after migration and the baseline cell-free area defined by the StenCell insert.

### 2.10. Apoptosis Assay

Apoptosis was evaluated using the Annexin V Apoptosis Detection Kit (BD Biosciences, San Diego, CA, USA, cat #556547; RRID: AB_2869082) and Propidium Iodide (PI; BD Biosciences, cat #556463; RRID: AB_2869075). SCC7 cells treated with or without diphenidol for 24 h were harvested, filtered (100 μm), and stained with Annexin V and PI for 30 min at 4 °C in the dark. Flow cytometry was performed using a FACSAria™ IIIu system (BD Biosciences), acquiring 10,000 events per sample. Annexin V^+^/PI^−^ and Annexin V^+^/PI^+^ populations represented early and late apoptotic/necrotic cells, respectively.

### 2.11. In Vivo Tumor Model

All animal experiments were performed in compliance with the ARRIVE 2.0 guidelines and institutional regulations for the care and use of laboratory animals. Experimental procedures were approved by the Ethics Review Committee for Animal Experimentation of the Health Sciences University of Hokkaido (protocol no. 23-048). An immunocompetent murine tumor model was established by subcutaneous implantation of SCC7 cells into genetically matched C3H/HeNSlc male mice (5 weeks old). These mice were purchased from Sankyo Labo Service Corporation, Inc. (Tokyo, Japan, RRID:MGI:2684584). The use of this strain ensured histocompatibility and minimized immune rejection.

Mice were included if they were clinically healthy, 5 weeks of age, and weighed 18–20 g at the start of the experiment. Animals were excluded if they exhibited pre-existing illness, visible skin lesions, abnormal behavior, or failure of tumor establishment following implantation. Mice that developed ulcerated tumors, showed distress, or displayed signs of pain or discomfort were humanely euthanized and excluded from the final analysis. All exclusions and reasons for removal were documented.

A total of 1 × 10^6^ SCC7 cells suspended in 100 µL of phosphate-buffered saline (PBS) were injected into the right dorsal flank of each mouse. Tumor growth and animal health were monitored at least three times per week by trained personnel who were blinded to treatment allocation. Animals were randomly assigned to receive subcutaneous injections of either diphenidol (103.7 mg/mL) or vehicle (saline) once daily from day 3 to day 9 post-inoculation, with 10 mice per group (*n* = 10). Diphenidol was dissolved in saline for in vivo administration to avoid potential toxicity associated with organic solvents such as DMSO. The group size was determined based on previous pilot studies and similar published models demonstrating consistent tumor growth kinetics, which allowed adequate detection of a 30% difference in tumor volume between groups with 80% power at a significance level of 0.05. Although no formal power calculation was conducted, this sample size was considered sufficient to ensure biological and statistical reliability while adhering to the 3Rs principle.

Tumor growth was monitored daily using caliper measurements, and tumor volume was calculated using a modified version of the ellipsoid formula: V = (4π/3) × [(*r*_1_/2 + *r*_2_/2)]^3^, where *r*_1_ and *r*_2_ represent the longitudinal and transverse radii, respectively [25,26]. On day 10 post-inoculation, mice were euthanized, and tumors were excised, weighed, and subjected to histological examination and flow cytometric analysis. Each in vivo experiment was independently repeated three times to ensure reproducibility.

### 2.12. Immunohistochemistry and TUNEL Assay

To assess tumor morphology and apoptosis in vivo, excised tumor tissues were fixed in 4% paraformaldehyde (in 0.1 M PBS, pH 7.4) at 4 °C for 24 h and subsequently embedded in paraffin (Fisher Scientific, Fair Lawn, NJ, USA). Longitudinal sections (4 μm thickness) were prepared, deparaffinized in xylene, rehydrated through a graded ethanol series, and stained with Mayer’s hematoxylin and eosin (H&E) solution (Muto Pure Chemicals Co., Tokyo, Japan) for histopathological evaluation.

Apoptotic cell death was evaluated using a terminal deoxynucleotidyl transferase-mediated dUTP nick end labeling (TUNEL) assay (Takara Bio, Otsu, Japan; cat #MK500). Deparaffinized sections were pretreated with proteinase K to expose nuclear content, followed by incubation with the TUNEL reaction mixture at 37 °C for 1 h. Detection was performed using an anti-fluorescein antibody and visualized with diaminobenzidine (DAB). Sections were then counterstained, dehydrated, permeabilized, and coverslipped. Cells with yellow-brown nuclear staining were considered TUNEL-positive and indicative of apoptosis.

Quantification of apoptotic cells was performed by counting TUNEL-positive nuclei at 40× magnification across four randomly selected fields per tumor section. The apoptotic index was expressed as the percentage of TUNEL-positive cells relative to the total number of cells in the observed fields: Apoptotic Index (%) = (TUNEL-positive cells/total cells) × 100.

### 2.13. Flow Cytometry of Tumor-Infiltrating Lymphocytes (TILs)

To characterize the immune cell composition within the tumor microenvironment, tumor-infiltrating lymphocytes (TILs) were isolated from excised tumors. Tumor tissues were finely minced (2–5 mm^3^) and enzymatically dissociated using Tumor and Tissue Dissociation Reagent (TTDR; BD Biosciences, cat #661563), following the manufacturer’s instructions. The resulting suspensions were filtered through 100 μm strainers (Corning, Corning, NY, USA; cat #431752), washed, and centrifuged to obtain single-cell suspensions.

Cells were stained with fluorophore-conjugated monoclonal antibodies targeting CD3, CD4, CD8, CD25, and Foxp3 (BD Pharmingen™) and analyzed by flow cytometry using a FACSAria™ IIIu system (BD Biosciences). Regulatory T cells (Tregs) were defined as CD4^+^CD25^+^Foxp3^+^. Data were analyzed using FlowJo software (Tree Star, Ashland, OR, USA, RRID: SCR_008520).

### 2.14. Statistical Analysis

All statistical analyses were performed using IBM SPSS Statistics software, version 26.0 (IBM Corp., Armonk, NY, USA; RRID:SCR_016479). Data are expressed as the mean ± standard deviation (SD) from at least three independent biological replicates unless otherwise stated. Prior to analysis, data distribution normality was verified using the Shapiro–Wilk test, and homogeneity of variances was evaluated using Levene’s test. Only datasets meeting these assumptions were analyzed using parametric tests; otherwise, appropriate non-parametric alternatives were applied. Comparisons between two independent groups (e.g., vehicle vs. diphenidol-treated samples) were analyzed using an unpaired two-tailed Student *t*-test, assuming independence and equal variances. For multiple group comparisons, a one-way ANOVA was performed with treatment concentration or time as a factor, followed by appropriate post hoc tests. Dunnett’s post hoc test was used for comparisons between each treatment group and the control group, whereas Tukey’s multiple comparison test was applied for pairwise comparisons among groups, including comparisons of histological grades and clinical stages in TMA analyses. Non-normally distributed data, such as qRT-PCR results, were analyzed using the Mann–Whitney U test, while categorical flow cytometry data were analyzed using the Chi-squared (χ^2^) test. A two-sided *p*-value < 0.05 was considered statistically significant, and *p* < 0.01 was regarded as highly significant. Representative graphs display the mean ± SD, and all experiments were independently repeated at least three times to ensure reproducibility.

## 3. Results

### 3.1. TAS2R38 Is Robustly Upregulated and Predominantly Localized to the Nucleus in Human Tongue OSCC

To define the pathological relevance of TAS2R38 in OSCC, we performed immunohistochemical analysis using surgically resected tumor tissues (*n* = 10) and an independent TMA cohort. In representative surgical specimens (Figure 1A–C), TAS2R38 expression was weak in histologically normal tongue epithelium at the surgical margin. In contrast, markedly increased immunoreactivity with striking nuclear localization was consistently observed in dysplastic lesions and invasive carcinoma cells infiltrating into the underlying connective tissue. Notably, this nuclear-dominant staining pattern was reproducible across nearly all examined specimens, indicating that nuclear localization of TAS2R38 represents a conserved feature of OSCC pathology. These observations suggest that TAS2R38 may function beyond a conventional membrane receptor, potentially acting as a regulator of transcriptional programs. However, the precise subcellular localization of TAS2R38 within the nucleus (e.g., nucleoplasm versus nuclear membrane) could not be determined based on immunohistochemical analysis. To further establish the clinicopathological significance of TAS2R38 expression, we analyzed tongue OSCC cases from the TMA cohort using pooled evaluable spots (*n* = 222). TAS2R38 expression was significantly elevated in OSCC tissues compared with normal tongue epithelium (one-way ANOVA followed by Tukey’s multiple comparison test, *p* < 0.05). Stratification by histological grade revealed that TAS2R38 expression was significantly increased in Grade I, Grade II, and Grade III tumors relative to normal tissue (Figure 1D). However, no significant difference was observed between Grade I and Grade II tumors, indicating that TAS2R38 upregulation is not a late event associated with progressive histological dedifferentiation, but rather represents an early and sustained molecular mechanistic alteration associated with malignant epithelial transformation. A similar pattern was observed across clinical stages. TAS2R38 expression was significantly higher in Stage I, Stage II, and Stage III tumors compared with normal tissue (Figure 1E). While no significant difference was detected between Stage I and Stage II tumors, Stage III tumors exhibited significantly higher expression than Stage II tumors, suggesting a further augmentation of TAS2R38 signaling in advanced disease. Importantly, the early elevation of TAS2R38 expression indicates that its dysregulation is not restricted to tumor progression but is initiated at early stages of tumorigenesis and maintained throughout disease advancement. Consistent with our immunohistochemical findings, in silico analyses using publicly available datasets further supported the clinical relevance of TAS2R38. Analysis of TCGA and GTEx datasets via GEPIA demonstrated that TAS2R38 expression was significantly elevated in HNSC tumor tissues compared with normal tissues (Supplementary Figure S1A). Kaplan–Meier survival analysis indicated no significant difference in overall survival between high and low TAS2R38 expression groups (Supplementary Figure S1B), suggesting that its prognostic value may be limited. In addition, analysis using UALCAN confirmed that TAS2R38 expression was higher in primary tumors than in normal tissues (Supplementary Figure S2A). Stage-wise analysis demonstrated that TAS2R38 expression varied across pathological stages (Stage I–IV), although no clear monotonic trend was observed (Supplementary Figures S1C and S2B). Furthermore, TAS2R38 expression showed variation according to nodal metastasis status (N0–N3), suggesting a potential but not definitive association with metastatic burden (Supplementary Figure S2C). Together, these findings reinforce the clinical and biological relevance of TAS2R38 in OSCC progression. Collectively, these findings identify TAS2R38 overexpression and predominant nuclear localization as robust and reproducible histopathological features of human OSCC. These data further implicate TAS2R38 as a potential regulator of tumor-associated transcriptional programs and highlight its relevance as a candidate biomarker and therapeutic target in OSCC.

### 3.2. Diphenidol Selectively Activates Intracellular Calcium Signaling in SCC7 Cells via IP_3_-Sensitive Ca^2+^ Stores

**Figure 2:**
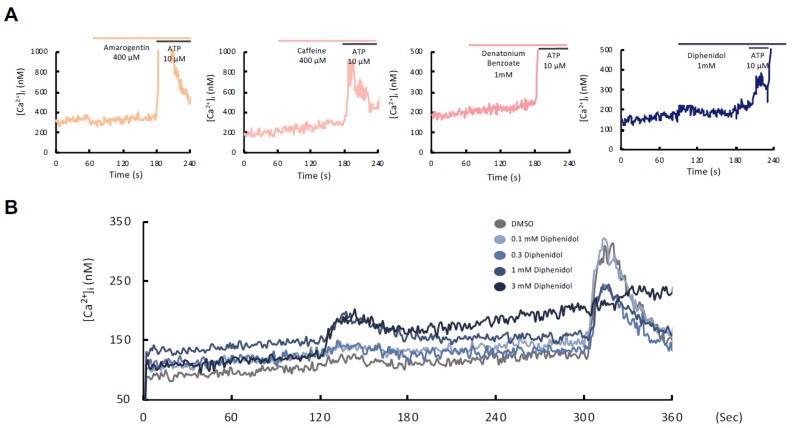

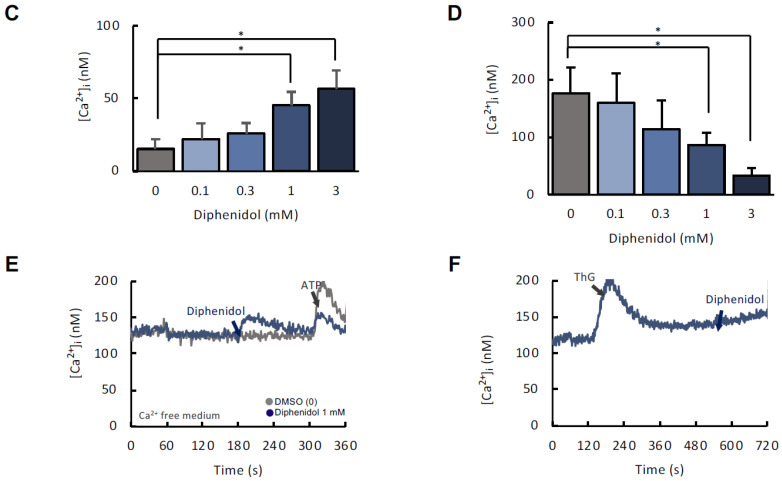
Diphenidol-induced intracellular Ca^2+^ signaling in SCC7 cells. (**A**) Among the four TAS2R agonists tested, only diphenidol elicited robust intracellular Ca^2+^ mobilization. (**B**) Diphenidol induced a biphasic increase in intracellular Ca^2+^ concentration ([Ca^2+^]ᵢ) in a dose-dependent manner. (**C**) Quantification demonstrated significantly elevated peak [Ca^2+^]ᵢ at 1 mM and 3 mM compared with the control. (**D**) Pretreatment with diphenidol attenuated ATP-induced Ca^2+^ release at 1 mM and 3 mM. (**E**) Diphenidol induced intracellular Ca^2+^ release in Ca^2+^-free medium, indicating that Ca^2+^ mobilization originated from intracellular stores. (**F**) Thapsigargin pretreatment abolished the diphenidol-induced Ca^2+^ response, confirming the involvement of endoplasmic reticulum (ER) Ca^2+^ stores. Data are representative of three independent experiments (* *p* < 0.05).

To determine functional Tas2r activity, we evaluated four bitter taste receptor agonists, amarogentin, caffeine, denatonium benzoate, and diphenidol, for their ability to induce intracellular Ca^2+^ mobilization. Among these compounds, diphenidol was the only agonist that induced a detectable increase in intracellular Ca^2+^ ([Ca^2+^]ᵢ) in SCC7 cells (Figure 2A). A biphasic, dose-dependent response was observed following treatment with 0.1–3 mM diphenidol (Figure 2B), with significantly elevated peak [Ca^2+^]ᵢ at 1 and 3 mM compared with controls (*p* < 0.05) (Figure 2C). Moreover, ATP-induced Ca^2+^ release was attenuated after diphenidol pretreatment at higher concentrations (Figure 2D). Diphenidol also triggered a transient increase in intracellular Ca^2+^ in Ca^2+^-free buffer (Figure 2E). This response was abolished by thapsigargin, an inhibitor of endoplasmic reticulum (ER) Ca^2+^-ATPase, suggesting that intracellular Ca^2+^ release was primarily derived from endoplasmic reticulum (ER) calcium stores (Figure 2F). All experiments were independently repeated in triplicate with consistent results. These findings indicate that diphenidol induces intracellular Ca^2+^ signaling, potentially mediated by TAS2rs in SCC7 cells, which may function as an upstream regulator of the observed transcriptional and functional changes.

### 3.3. Diphenidol Modulates Transcriptional Profiles Associated with Immune Regulation and Apoptosis

**Figure 3:**
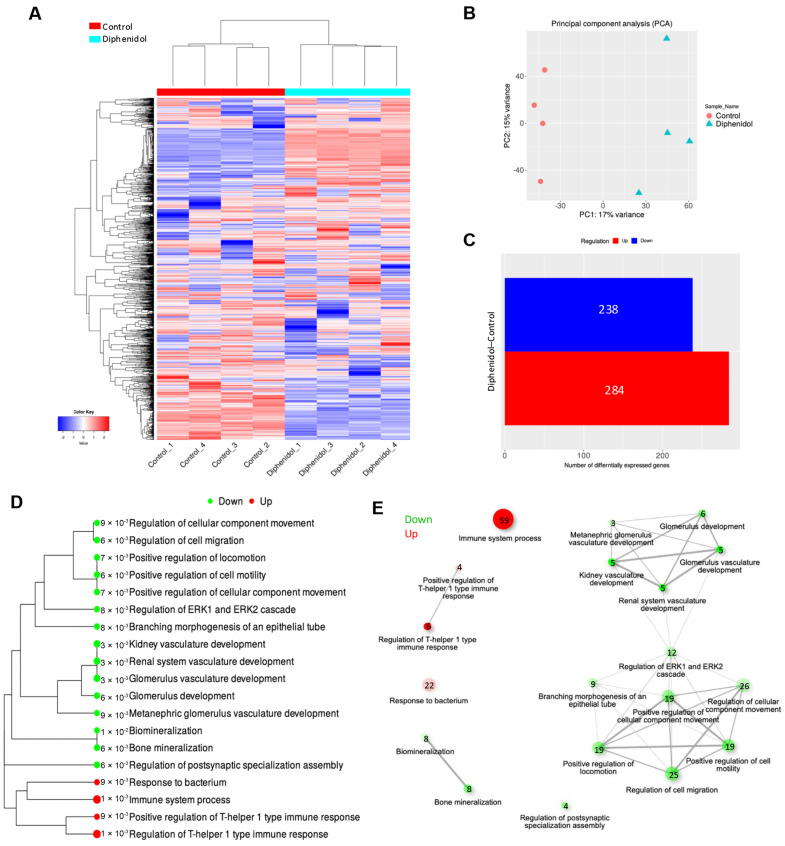
Transcriptomic analysis of SCC7 cells following treatment with 0.1 mM diphenidol for 24 h. (**A**) Heatmap showing hierarchical clustering of differentially expressed genes (DEGs) between control and diphenidol-treated cells. (**B**) Principal component analysis (PCA) demonstrating distinct clustering between control and treatment groups. (**C**) Volcano plot identifying 284 upregulated and 238 downregulated genes (>2-fold change). (**D**) Gene ontology (GO) enrichment analysis indicating enrichment of multiple biological processes. Although immune-related pathways were not significantly enriched, several genes associated with immune regulation were included in the analysis. (**E**) Network visualization of GO terms illustrating the relationships among enriched biological processes, with node size reflecting gene counts and color indicating upregulated (red) or downregulated (green) pathways. Results are based on quadruplicate biological samples (*n* = 4 per group), and representative data are shown.

To further investigate the transcriptional changes associated with diphenidol treatment, we performed RNA sequencing analysis. RNA-seq analysis revealed significant gene expression changes in SCC7 cells treated with 0.1 mM diphenidol for 24 h. Hierarchical clustering and principal component analysis (PCA) demonstrated a clear separation between diphenidol-treated and control groups (Figure 3A,B). A volcano plot identified 284 upregulated and 238 downregulated genes with a fold change greater than two (Figure 3C). Gene Ontology (GO) enrichment analysis indicated that differentially expressed genes were enriched in immune-related pathways and apoptotic processes, including genes associated with the Th1-type immune response (Figure 3D,E). These transcriptomic findings were further validated by qRT-PCR analysis. The mRNA levels of Tas2r105, Tas2r137, Il1rl1, and Lzts2 were significantly increased in the diphenidol-treated group compared with the control (*p* < 0.05), consistent with the RNA-seq results. In contrast, Heca and Sipa1l1 mRNA levels showed no significant differences between groups (Figure S3). All RNA-seq experiments were performed using four biologically independent replicates (*n* = 4).

### 3.4. Diphenidol Inhibits Proliferation and Migration and Induces Apoptosis in SCC7 Cells

**Figure 4:**
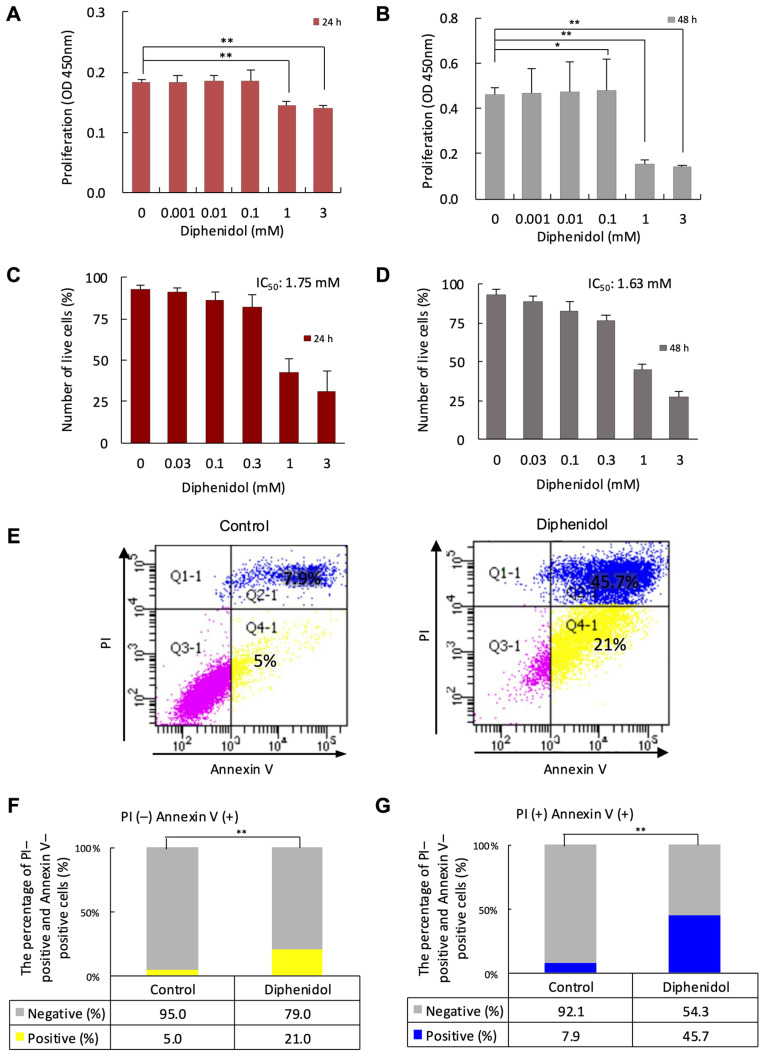
Diphenidol suppresses proliferation and induces apoptosis in SCC7 cells. (**A**,**B**) WST-1 assays demonstrated dose-dependent inhibition of SCC7 cell proliferation at 24 and 48 h following diphenidol treatment. (**C**,**D**) Trypan blue exclusion assays confirmed cytotoxic effects of diphenidol, with an estimated IC_50_ of approximately 1.75 mM. (**E**) Representative flow cytometry plots showing Annexin V/PI staining of SCC7 cells following diphenidol treatment. (**F**) Quantification of early apoptotic cells (Annexin V^+^/PI^−^) demonstrating a significant increase in diphenidol-treated cells. (**G**) Quantification of late apoptotic cells (Annexin V^+^/PI^+^) also showing a significant increase after diphenidol treatment. All results were confirmed in three biologically independent experiments, and representative data are shown (* *p* < 0.05, ** *p* < 0.01).

In WST-1 assays, SCC7 cell proliferation was significantly inhibited at 24 and 48 h following diphenidol treatment at concentrations ≥ 0.1 mM (Figure 4A,B; *p* < 0.05). Trypan blue exclusion assays confirmed the cytotoxic effects of diphenidol in a dose-dependent manner, with an estimated IC_50_ of approximately 1.75 mM at 24 h (Figure 4C,D). In addition, diphenidol significantly reduced SCC7 cell migration at concentrations ≥ 0.1 mM after 24 h (*p* < 0.05; Figure S4A,B). Apoptosis analysis using Annexin V/PI staining demonstrated a significant increase in early apoptotic cells (Annexin V^+^/PI^−^) (*p* < 0.01; Figure 4F) and late apoptotic cells (Annexin V^+^/PI^+^) (*p* < 0.01; Figure 4G) following diphenidol treatment (Figure 4E). These findings indicate that diphenidol induces apoptosis in SCC7 cells, as evidenced by increased Annexin V-positive populations. All experiments were independently repeated at least three times with consistent results.

### 3.5. Diphenidol Suppresses Tumor Growth and Enhances Apoptosis In Vivo

**Figure 5:**
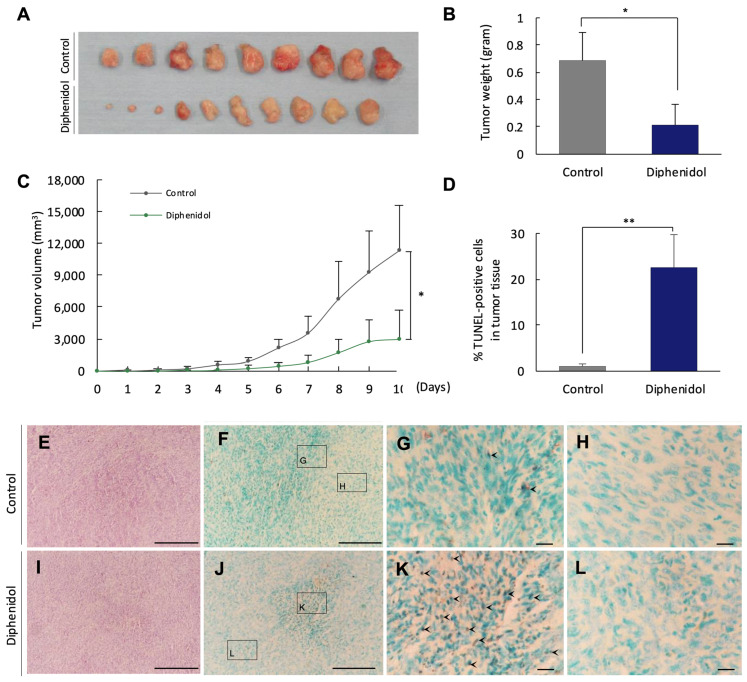

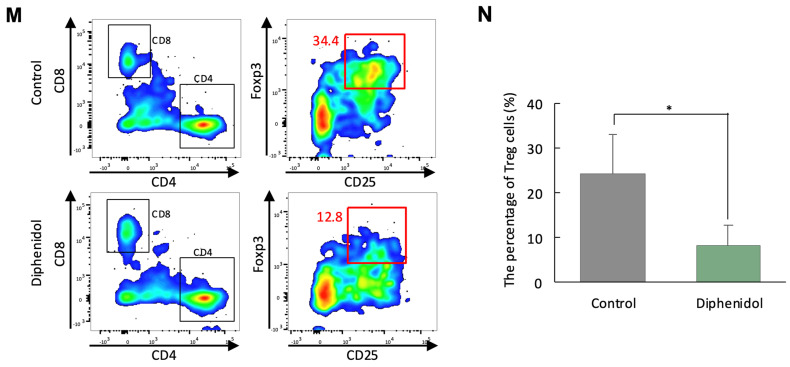
Diphenidol inhibits tumor growth and promotes apoptosis in vivo. (**A**) Representative images of excised tumors on day 10 after SCC7 cell implantation. (**B**,**C**) Tumor volume and tumor weight were significantly reduced in the diphenidol-treated group compared with the control group. (**D**) Quantification of apoptotic cells in tumor tissues by TUNEL assay showing significantly increased apoptosis in the diphenidol-treated group. (**E**–**L**) Representative TUNEL-stained tumor sections from control and diphenidol-treated groups. (**G**,**K**) Arrowheads indicate TUNEL-positive apoptotic nuclei within tumor regions. (**M**) Flow cytometry analysis of tumor-infiltrating regulatory T cells (CD4^+^CD25^+^Foxp3^+^ Tregs). Tumor-infiltrating lymphocytes were initially gated based on CD4 and CD8 expression (black boxes), followed by identification of CD4^+^CD25^+^Foxp3^+^ Tregs within the CD4^+^ population (red boxes). Treg cells were defined as CD4^+^CD25^+^Foxp3^+^ T cells and quantified as a percentage of CD3^+^ tumor-infiltrating lymphocytes. (**N**) Quantification demonstrating significantly reduced Treg infiltration in diphenidol-treated tumors. A trend toward an increased CD8^+^/Treg ratio was also observed (Supplementary Figure S5B). Data are representative of three independent experiments. For in vivo tumor growth analyses (**A**–**L**), n = 10 mice per group were used. Flow cytometry analyses (**M**,**N**) were performed using representative tumor samples (*n* = 4–5 per group). (* *p* < 0.05, ** *p* < 0.01).

To evaluate the in vivo antitumor efficacy of diphenidol, we employed a syngeneic mouse model established by subcutaneous implantation of SCC7 tumor cells. For these in vivo experiments, 10 mice per group were used (*n* = 10). Daily administration of diphenidol (103.7 mg/kg) significantly reduced tumor volume and tumor weight by day 10 compared with the vehicle-treated group (*p* < 0.05; Figure 5A–C). TUNEL staining revealed a marked increase in apoptotic cells in tumors from diphenidol-treated mice (*p* < 0.01; Figure 5D). Representative histological images showed numerous TUNEL-positive nuclei within tumor tissues, particularly at the invasive tumor front (arrowheads; Figure 5G,K). These findings indicate that diphenidol suppresses tumor progression in vivo, at least in part through the induction of tumor cell apoptosis. All experiments were independently repeated in three biological replicates with consistent results.

### 3.6. Diphenidol Modulates the Tumor Immune Microenvironment by Reducing Regulatory T Cell Infiltration

Flow cytometry analysis of tumor-infiltrating lymphocytes revealed a significant decrease in CD4^+^CD25^+^Foxp3^+^ regulatory T cells (Tregs) in diphenidol-treated mice compared with vehicle-treated controls (*p* < 0.05; Figure 5M,N). For this analysis, representative tumor samples were harvested on day 10 from the control group (*n* = 4) and the diphenidol-treated group (*n* = 5), selected from the in vivo experimental cohort (*n* = 10 per group). Statistical analysis was performed using the Mann–Whitney U test (*p* = 0.0159). Error bars represent median ± standard error. The gating strategy used for T cell subset identification is shown in Figure S5A. Although no statistically significant difference was observed in CD8^+^ T cells, a trend toward an increased CD8^+^/Treg ratio was noted in the diphenidol-treated group (Supplementary Figure S5B). These results indicate that diphenidol reduces Treg infiltration and thereby modulates the tumor immune microenvironment, suggesting that TAS2R-mediated Ca^2+^ signaling may be involved in tumor immunometabolic regulation. To further explore the relationship between TAS2R38 and tumor immunity, we performed in silico correlation analyses using TCGA datasets. TAS2R38 expression showed minimal correlation with FOXP3 and CD8A expression in HNSC samples (Supplementary Figure S6), indicating that any association with immune-related markers is likely limited. In addition, mapping of differentially expressed genes onto the T cell receptor signaling pathway revealed partial modulation of immune-related signaling components, although this pathway was not significantly enriched in KEGG analysis (Supplementary Figure S7). These findings suggest that the immunomodulatory effects observed in vivo may not be directly reflected at the transcriptomic level and may involve context-dependent or indirect mechanisms. These results were consistent across multiple independent experiments.

## 4. Discussion

Taste alterations during cancer treatment represent a clinically significant adverse event that directly affects patients’ nutritional status and quality of life. These changes are frequently observed as side effects of chemotherapy, radiotherapy, and targeted therapies, and are particularly pronounced in patients receiving chemotherapy, where they contribute to oral functional decline and impaired dietary intake [27,28]. Increasing evidence suggests that these alterations may involve the dysregulation of taste receptors, particularly bitter taste receptors (TAS2Rs), which are now recognized to play roles beyond gustation, including in cancer biology [4,29].

In the present study, immunohistochemical and TMA analyses demonstrated that TAS2R38 expression is increased in OSCC tissues compared with normal tongue epithelium and is associated with histological grade and clinical stage. Furthermore, in surgically resected tongue OSCC specimens, TAS2R38 exhibited predominant nuclear localization, representing a reproducible histopathological feature linked to malignant epithelial transformation. Collectively, these findings indicate that TAS2R38 expression is already elevated at early stages of tumorigenesis, maintained throughout disease progression, and further augmented in advanced-stage tumors, suggesting that its upregulation represents an early and sustained molecular alteration during OSCC progression.

Given that the expression and functional activity of TAS2Rs are dynamically regulated in a stage- and microenvironment-dependent manner, we further investigated the expression and functional roles of Tas2r subtypes in murine models. The expression of murine Tas2r subtypes has been reported in various cancer types [22,29], with additional studies further characterizing their roles in tumor biology and signaling across multiple cancer types [30,31,32,33], as well as in tumor progression and tumor microenvironment interactions [34,35,36,37,38,39], and their role in head and neck squamous cell carcinoma (HNSCC) was first demonstrated in a mouse model by Carey et al. [20]. As members of the G protein-coupled receptor (GPCR) family, TAS2Rs primarily signal through intracellular calcium pathways. Accordingly, bitter compounds are widely used as agonists to activate Tas2r-mediated intracellular calcium ([Ca^2+^]_i_) signaling.

Among the compounds tested, diphenidol, a synthetic agonist that activates 15 human and 6 murine TAS2Rs, was the only agent that induced a significant increase in intracellular Ca^2+^ levels in SCC7 cells, whereas amarogentin, caffeine, and denatonium benzoate failed to elicit a response. This calcium elevation is mediated through the canonical GPCR-PLC-IP3 signaling cascade, leading to calcium release from the endoplasmic reticulum (ER), which regulates diverse cellular processes including proliferation, apoptosis, and stress responses [40,41,42]. Although previous studies have examined multiple TAS2R agonists, diphenidol was selected in this study based on its robust ability to induce intracellular Ca^2+^ signaling in our experimental system and its clinical applicability. This focused approach enabled a detailed evaluation of TAS2R-mediated effects on tumor growth and the tumor immune microenvironment in OSCC. Furthermore, RNA sequencing analysis revealed that diphenidol treatment significantly upregulated genes associated with immune modulation and tumor suppression, including Il1rl1 and Lzts2 (Table 1). These genes were selected for validation based on their known involvement in immune regulation and tumor-suppressive pathways, their biological relevance in cancer, and their consistent and robust upregulation with relatively high expression changes in the RNA-seq dataset. IL1RL1, a component of the IL-33 signaling axis, is involved in the regulation of inflammation and cancer risk [43,44], whereas LZTS2 is a transcriptional regulator that suppresses PI3K/AKT signaling and is frequently downregulated in various cancers [45,46]. Additionally, although only a subset of genes was validated, pathway-level analysis suggested potential involvement of immune-related and apoptosis-associated signaling pathways, even though no statistically significant enrichment was observed, supporting the biological relevance of the transcriptomic findings. These findings suggest that diphenidol-induced signaling may influence gene networks involved in immune responses and tumor suppression. These molecular observations are consistent with previously reported antitumor effects of TAS2Rs. Activation of TAS2R4 and TAS2R14 has been shown to exert antiproliferative and pro-apoptotic effects in breast and ovarian cancer models [47]. Carey et al. [20] demonstrated that bitter agonist-induced nuclear calcium signaling suppresses cellular metabolism in HNSCC. In our syngeneic OSCC mouse model, systemic administration of diphenidol significantly reduced tumor volume and weight and increased apoptotic cell death, as confirmed by TUNEL staining. Notably, flow cytometric analysis revealed a marked reduction in tumor-infiltrating CD4^+^CD25^+^Foxp3^+^ regulatory T cells (Tregs), indicating attenuation of the immunosuppressive tumor microenvironment. Although no statistically significant increase in CD8^+^ T cell infiltration was observed, a trend toward an increased CD8^+^/Treg ratio was noted, suggesting a partial shift toward a more immunologically active tumor microenvironment. Collectively, these in vivo findings suggest that diphenidol partially alleviates immunosuppression and highlight a role for TAS2R-mediated Ca^2+^ signaling in immune modulation. However, in silico analyses using TCGA datasets demonstrated that TAS2R38 expression showed minimal correlation with FOXP3 and CD8A expression, indicating that the immunomodulatory effects observed in vivo may not be directly reflected at the transcriptomic level and may involve context-dependent or indirect mechanisms (Supplementary Figure S6). These findings underscore the complexity of TAS2R-mediated immune regulation and suggest that tumor–immune interactions may be influenced by cellular context rather than gene expression alone. In addition, Wei et al. [21] reported that TAS2R14 agonists induce apoptosis in papillary thyroid carcinoma and that high TAS2R14 expression is associated with favorable prognosis.

In contrast to previous reports focusing primarily on other TAS2R subtypes or tumor contexts, our results indicate that TAS2R38 expression is elevated from early stages of tumorigenesis, maintained throughout disease progression, and further enhanced in advanced disease, suggesting that TAS2R function may vary depending on tumor type and stage. These findings support the possibility that TAS2R agonists such as diphenidol may be particularly effective in advanced OSCC. Furthermore, TAS2Rs have been implicated in the regulation of tumor immune landscapes and stromal interactions. Tong et al. [48] demonstrated that TAS2R gene expression profiles stratify colorectal cancer patients into molecular subtypes with distinct immune characteristics and prognoses, with high TAS2R expression associated with enhanced immune infiltration and favorable outcomes. These findings are consistent with our observations and support the potential of TAS2Rs as both biomarkers and therapeutic targets.

Similarly, Hung et al. [49] identified TAS2R9 as a cancer-associated fibroblast (CAF)-selective marker in pancreatic ductal adenocarcinoma and demonstrated that TAS2R-targeted nanoparticle delivery to the stromal compartment resulted in significant tumor suppression. These findings highlight the multifaceted roles of TAS2Rs in both tumor cells and the tumor microenvironment.

Given that intracellular calcium signaling enhances T-cell receptor activation and suppresses Treg-mediated immunosuppression, these results provide a mechanistic basis linking TAS2R activation to immune modulation. These immunomodulatory effects are particularly relevant in the context of the limited efficacy of immune checkpoint inhibitors (ICIs), such as nivolumab, in platinum-resistant recurrent or metastatic HNSCC [50]. However, it remains unclear whether the observed changes in the tumor immune microenvironment are a direct consequence of TAS2R activation or secondary to tumor growth suppression. Given that tumor burden itself can influence immune cell composition, these findings may reflect both direct and indirect mechanisms. Although combination therapy was not directly evaluated in this study, the observed reduction in Treg infiltration and remodeling of the tumor immune microenvironment suggest that TAS2R agonists may complement existing immunotherapeutic strategies. Taken together, our findings demonstrate that TAS2R agonists such as diphenidol exert antitumor effects through both direct tumor cell inhibition and modulation of the tumor immune microenvironment. Similar findings have been reported in other cancer types, including thyroid and colorectal cancers [21,48], supporting the potential of TAS2Rs as promising therapeutic targets and biomarkers in cancer biology. Importantly, our results further suggest that TAS2R-mediated Ca^2+^ signaling represents a novel mechanistic link between tumor progression and immune regulation, integrating tumor-intrinsic and microenvironmental effects. This study has several limitations. Diphenidol is a non-selective TAS2R agonist, and the present study did not include receptor-specific validation (e.g., TAS2R38 knockdown), which limits mechanistic specificity; therefore, the specific contribution of TAS2R38 to the observed effects requires further investigation using loss-of-function approaches. In addition, in vivo immune analysis was primarily limited to Treg populations, and broader immune profiling remains to be explored. Furthermore, transcriptomic findings were validated only at the mRNA level without protein-level confirmation. Future studies using receptor-specific approaches and comprehensive immune profiling will be required to further elucidate the role of TAS2R signaling in tumor immunity. These findings provide a rationale for further investigation of TAS2R-targeted strategies in combination with existing cancer therapies.

## 5. Conclusions

This study identifies TAS2R signaling as a previously underappreciated regulator of oral squamous cell carcinoma (OSCC) progression and tumor immunity, and demonstrates that the TAS2R agonist diphenidol exerts antitumor effects through both tumor-intrinsic mechanisms and modulation of the tumor immune microenvironment. Mechanistically, diphenidol activates intracellular calcium signaling, enhances the expression of immune-regulatory and tumor-suppressive genes, and suppresses tumor cell proliferation and migration in vitro. Consistently, in vivo administration of diphenidol increases apoptotic cell death and reduces regulatory T-cell infiltration, suggesting partial remodeling of the tumor immune microenvironment. Importantly, given that diphenidol is a clinically approved drug with an established safety profile, these findings provide a strong rationale for drug-repurposing strategies targeting TAS2R signaling in cancer therapy. Collectively, TAS2R-mediated calcium signaling represents a novel mechanistic axis linking tumor progression and immunoregulation and may serve as a promising therapeutic target in OSCC. Further studies are warranted to elucidate downstream molecular mechanisms and to evaluate the therapeutic potential of TAS2R agonists, particularly in combination with existing treatments, including immune checkpoint inhibitors in resistant HNSCC. These findings provide a basis for future translational studies aimed at integrating TAS2R-targeted strategies into current therapeutic frameworks.

## Figures and Tables

**Table 1:** The differentially expressed genes (DEGs) most up- and down-regulated in response to treatment with the Tas2r agonist diphenidol.

Gene ID	Symbol	Description	log_2_ Fold Change	Adj *p* Values
**Upregulated Differentially Expressed Gene**				
ENSMUSG00000026069	Il1rl1	interleukin 1 receptor-like 1	5.702127071	2.80 × 10^−7^
ENSMUSG00000039879	Heca	hdc homolog, cell cycle regulator	6.528040111	2.25 × 10^−5^
ENSMUSG00000050776	Olfr1317	olfactory receptor family 4 subfamily F member 47	6.604364684	0.01636834
ENSMUSG00000021646	Mccc2	methylcrotonoyl-CoA carboxylase 2	6.635359028	0.00011617
ENSMUSG00000043909	Trp53bp1	transformation-related protein 53 binding protein 1	6.789957746	0.00026009
ENSMUSG00000047428	Dlk2	delta-like non-canonical Notch ligand 2	6.797139793	3.78 × 10^−5^
ENSMUSG00000047109	Cldn14	claudin 14	6.953696549	0.0995777
ENSMUSG00000048279	Sacs	sacsin	6.972006267	1.30 × 10^−5^
ENSMUSG00000015090	Ptgds	prostaglandin D2 synthase	7.07153037	0.00235089
ENSMUSG00000098650	Commd1b	COMM domain-containing protein 1	7.607206741	0.05875312
ENSMUSG00000031799	Tpm4	tropomyosin 4	9.192198312	2.50 × 10^−6^
ENSMUSG00000035342	Lzts2	leucine zipper, putative tumor suppressor 2	9.842655723	4.03 × 10^−7^
**Downregulated differentially expressed gene**				
ENSMUSG00000037716	Ccdc33	coiled-coil domain-containing 33	−7.892024778	0.05173466
ENSMUSG00000042700	Sipa1l1	signal-induced proliferation-associated 1 like 1	−7.717187171	2.65 × 10^−6^
ENSMUSG00000023505	Cdca3	cell division cycle-associated 3	−7.499259915	4.91 × 10^−6^
ENSMUSG00000010311	Optc	opticin	−6.858924507	0.09314864
ENSMUSG00000072964	Bhlhb9	basic helix-loop-helix domain-containing, class B9	−6.83308327	3.60 × 10^−5^
ENSMUSG00000032254	Kif23	kinesin family member 23	−6.815479484	8.94 × 10^−6^
ENSMUSG00000027646	Src	Rous sarcoma oncogene	−6.526260407	0.00029274
ENSMUSG00000030849	Fgfr2	fibroblast growth factor receptor 2	−6.048728642	0.00018604
ENSMUSG00000095909	Zfp997	zinc finger protein 997	−5.941494813	0.02891211
ENSMUSG00000063275	Hacd1	3-hydroxyacyl-CoA dehydratase 1	−5.803498901	1.28 × 10^−24^

## Data Availability

The RNA-seq data generated in this study have been deposited in the DDBJ BioProject database under accession number PRJDB39286 (https://ddbj.nig.ac.jp/resource/bioproject/PRJDB39286, accessed on 30 March 2026). All other data supporting the findings of this study are included in the published article.

## Supplementary Materials

The following supporting information can be downloaded at: https://www.mdpi.com/article/10.3390/cancers18101527/s1; Figure S1: Expression and prognostic relevance of TAS2R38 in HNSC; Figure S2: Clinicopathological association of TAS2R38 expression in HNSC; Figure S3: Validation of RNA-seq data by qRT-PCR; Figure S4: Effect of diphenidol on SCC7 cell migration; Figure S5: Flow cytometry analysis of tumor-infiltrating T cell subsets Figure S6: Correlation of TAS2R38 with immune-related markers in HNSC; Figure S7: Mapping of differentially expressed genes onto the T cell receptor signaling pathway; Table S1: Primer sequences of genes used for quantitative reverse transcription PCR.

## Abbreviations

TAS2R	Taste receptor type 2 (bitter taste receptor)
OSCC	Oral squamous cell carcinoma
ICI	Immune checkpoint inhibitor
TME	Tumor microenvironment
GPCR	G protein-coupled receptor
IP_3_	Inositol 1,4,5-trisphosphate
SHH	Sonic hedgehog
ATP	Adenosine 5′-triphosphate disodium salt hydrate
PI3K/AKT	Phosphatidylinositol 3-kinase/protein kinase B
MAPK	Mitogen-activated protein kinase
ER	Endoplasmic reticulum
TMA	Tissue microarray
TAS2R38	Taste receptor type 2 member 38
SCC7	Murine squamous cell carcinoma cell line
ATCC	The American Type Culture Collection
RNA-seq	RNA sequencing
GO	Gene ontology
qRT-PCR	Quantitative reverse transcription polymerase chain reaction
TILs	Tumor-infiltrating lymphocytes
