# Supplementary material for: Activation of TAS2R Signaling by Diphenidol Suppresses Tumor Growth and Remodels the Tumor Immune Microenvironment in Oral Squamous Cell Carcinoma
Source: Cancers (Basel). 2026 May 9;18(10):1527. doi: 10.3390/cancers18101527 (PMC13204434; doi:10.3390/cancers18101527)
Supplement: Supplementary file 1 [file cancers-18-01527-s001.zip › cancers-4258036-supplementary.pdf]

Supplementary figures

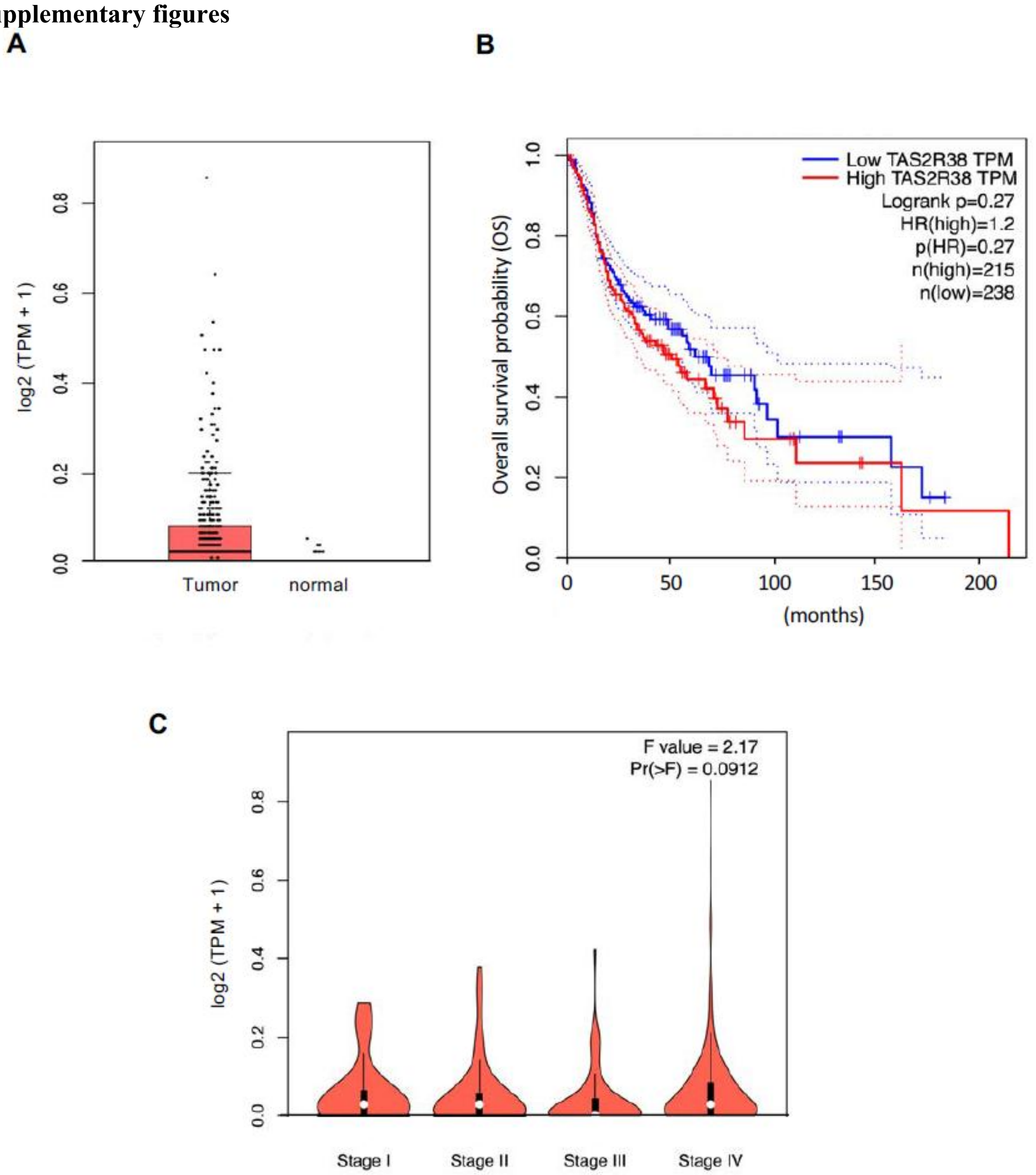

Figure S1. Expression and prognostic relevance of TAS2R38 in HNSC. (A) Differential expression of TAS2R38 between tumor (n = 519) and normal tissues (n = 44) in HNSC derived from TCGA and GTEx datasets using GEPIA. Expression values are presented as log2 (TPM + 1). (B) Kaplan–Meier overall survival analysis stratified by median TAS2R38 expression. Patients were divided into high and low expression groups. Log-rank test p-value and hazard ratio (HR) are indicated, whereas the dotted lines represent the 95% confidence intervals of the Kaplan–Meier survival curves. (C) Stage-wise expression analysis of TAS2R38 across pathological stages (Stage I–IV) in HNSC. Statistical significance was evaluated using one-way ANOVA.

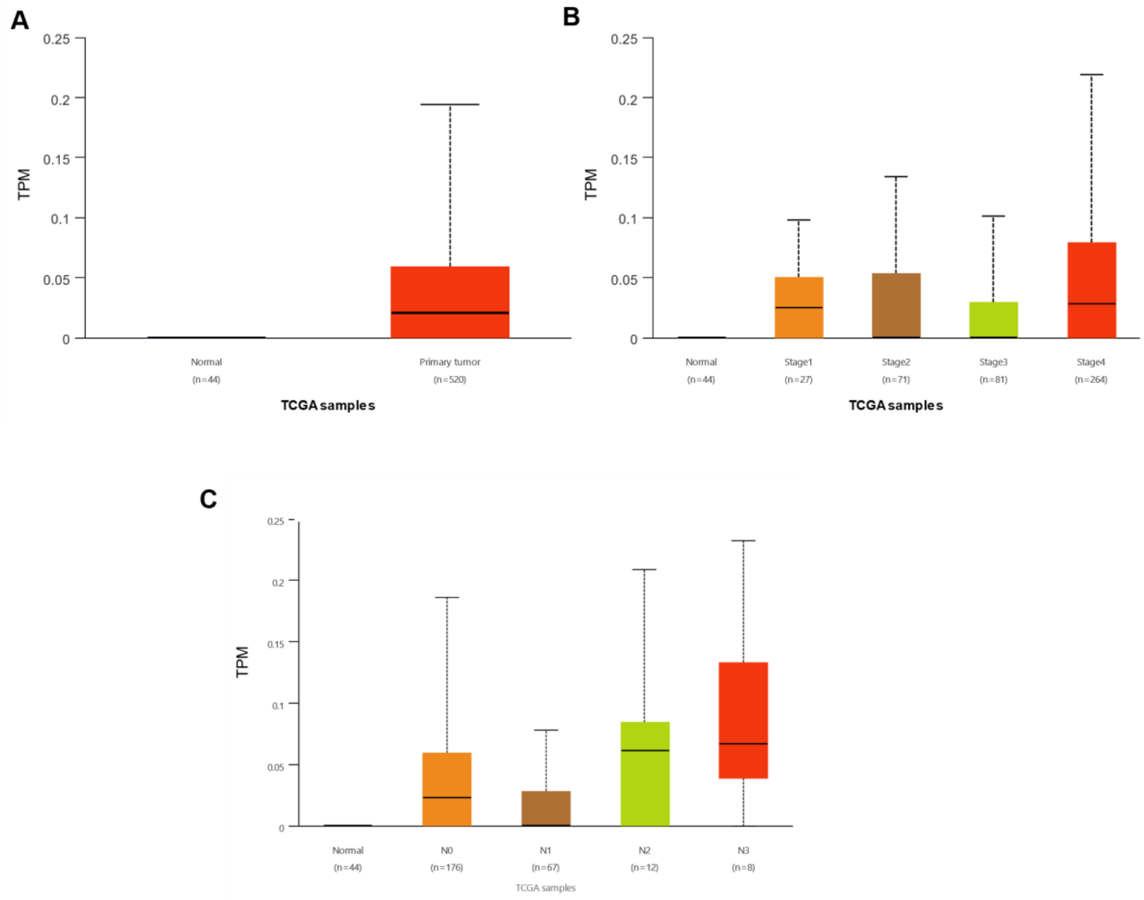

Figure S2. Clinicopathological association of TAS2R38 expression in HNSC. (A) Comparison of TAS2R38 expression between normal tissues (n = 44) and primary tumors (n = 520) in HNSC using TCGA data accessed via UALCAN. (B) Expression of TAS2R38 stratified by pathological stage (Stage I–IV). (C) Expression of TAS2R38 according to nodal metastasis status (N0–N3).

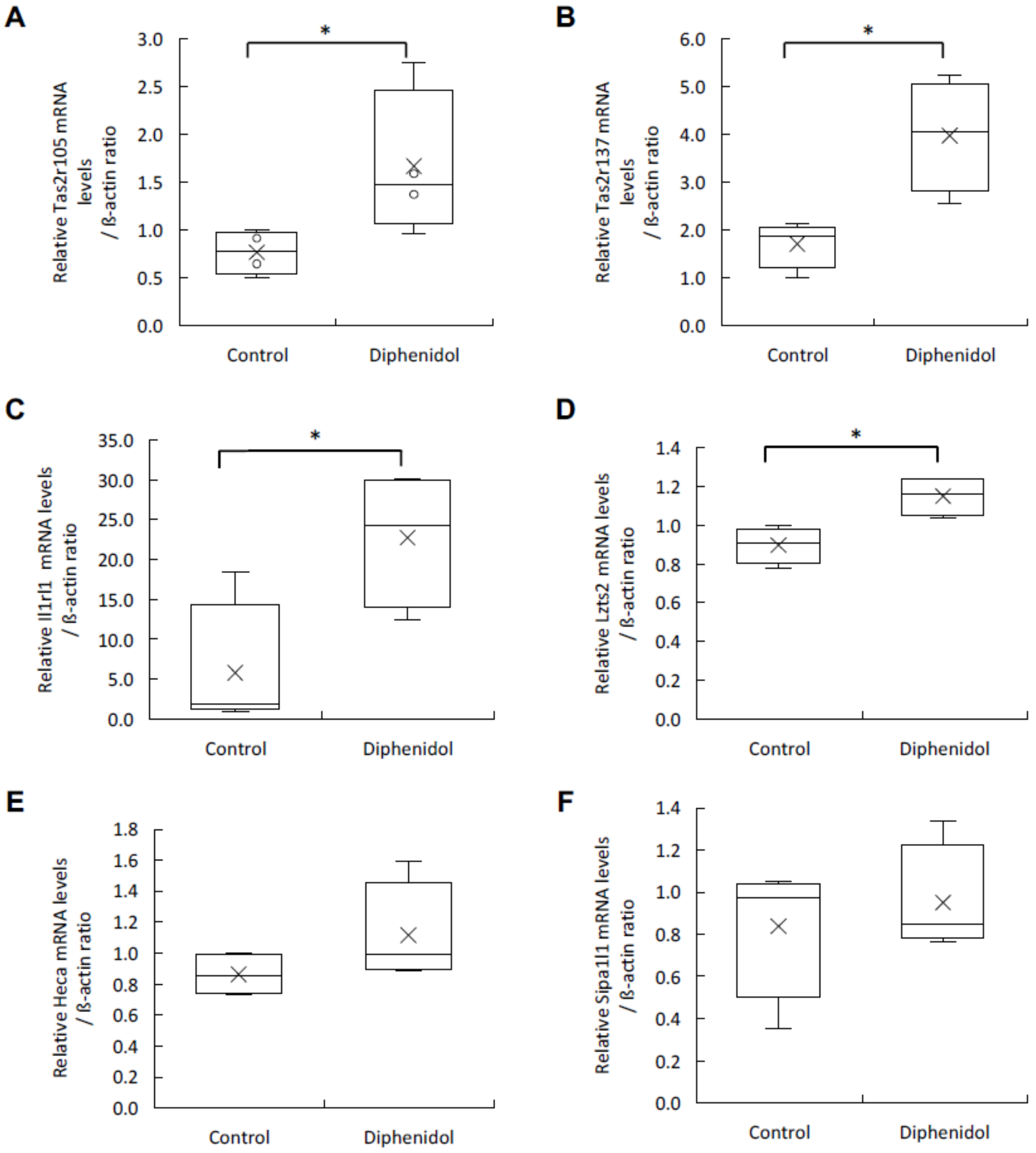

Figure S3. Validation of RNA-seq data by qRT-PCR. qRT-PCR analysis demonstrated that the mRNA expression levels of Tas2r105 (A), Tas2r137 (B), Il1rl1 (C), and Lzts2 (D) were significantly increased following diphenidol treatment (0.1 mM) compared with the control (*p < 0.05). In contrast, Heca (E) and Sipa1l1 (F) showed an increasing trend without reaching statistical significance (ns). Data represent mean ± SD from three independent experiments. Statistical analysis was performed using the Mann–Whitney U test (*$p < 0.05$).

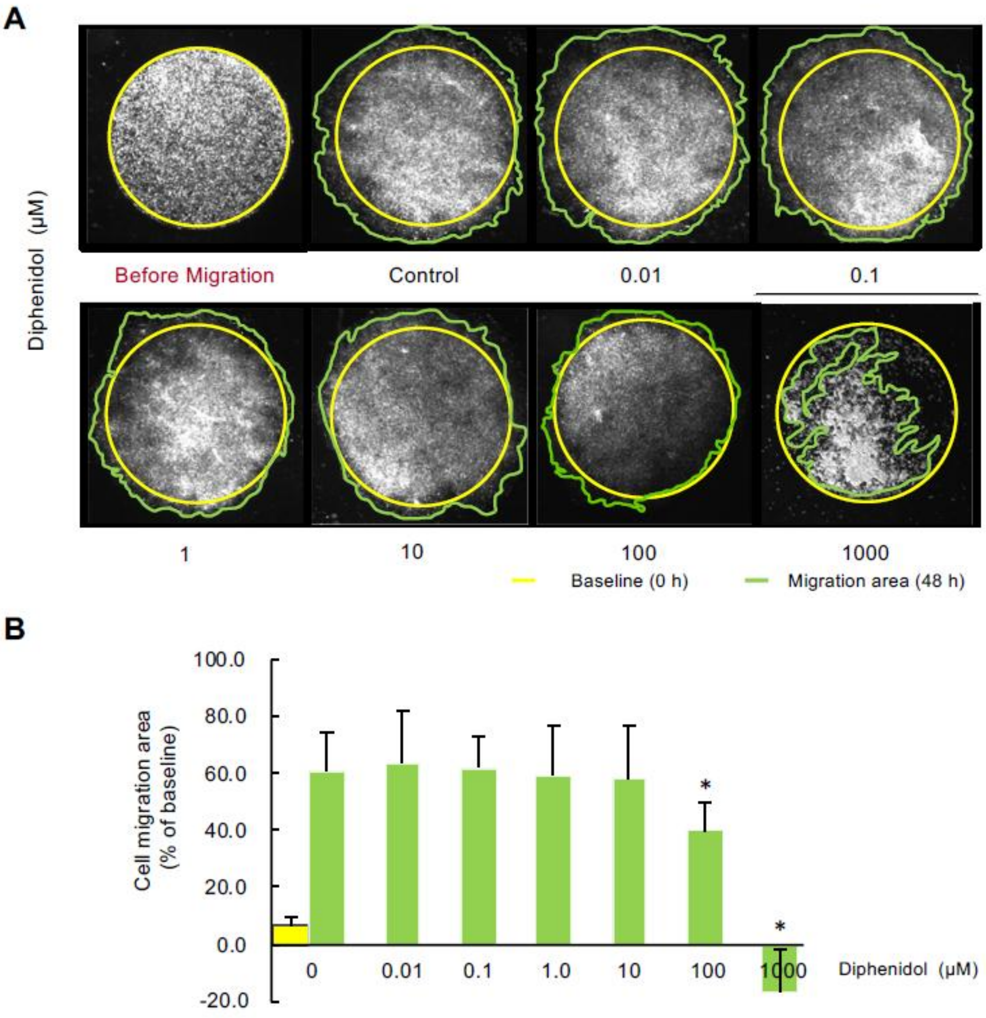

Figure S4. Effect of diphenidol on SCC7 cell migration. Representative images of SCC7 cell migration (A) and quantitative analysis of migrated area (B) following diphenidol treatment are shown. Migration was significantly reduced at concentrations ≥ 0.1 mM (*p < 0.05). Data represent mean ± SD from three independent experiments.

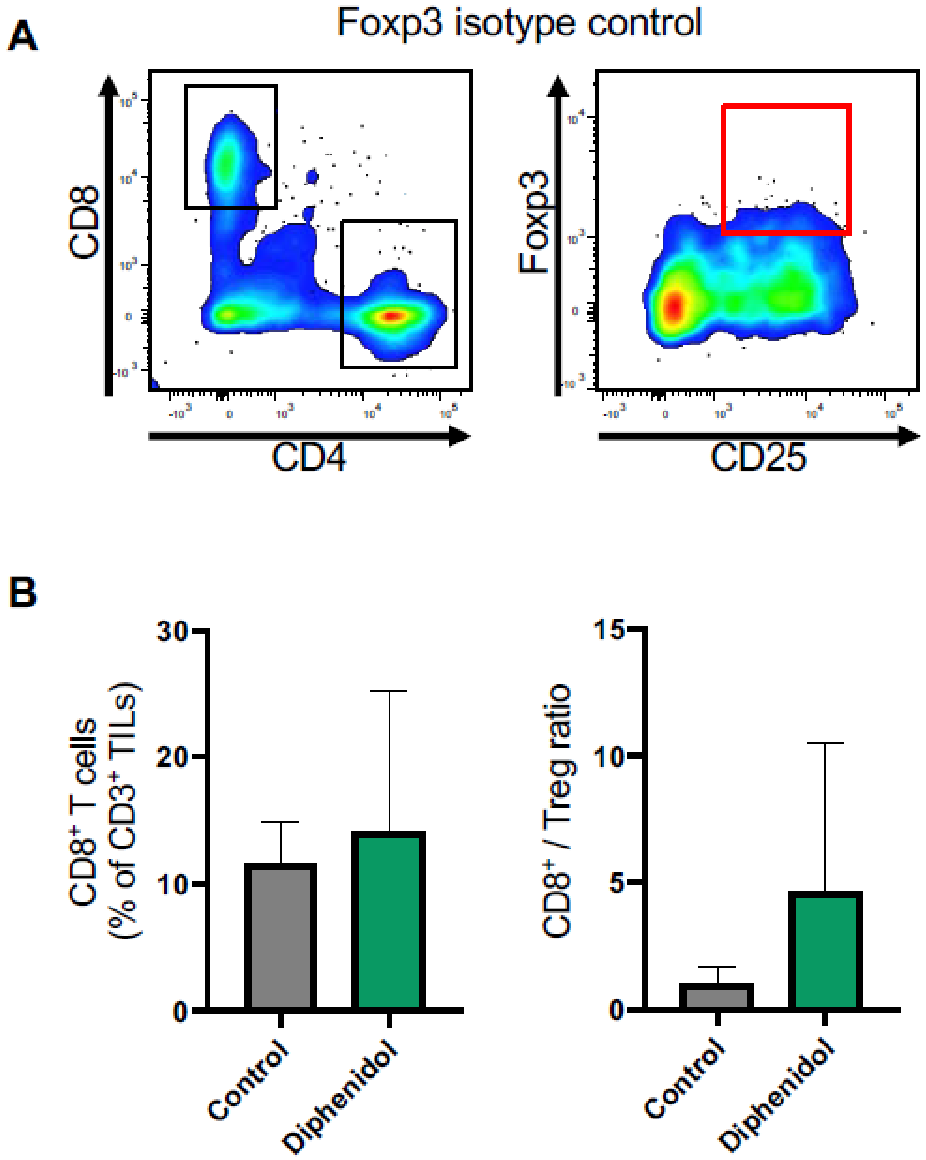

Figure S5. Flow cytometry analysis of tumor-infiltrating T cell subsets. (A) Flow cytometry gating strategy for Foxp3$^+$ cells using an isotype control. Representative plots are shown to define CD4$^+$CD25$^+$Foxp3$^+$ regulatory T cells (Tregs). CD4$^+$ and CD8$^+$ T-cell subsets were initially defined based on CD4 versus CD8 expression profiles (black boxes). Foxp3 expression was subsequently evaluated within the CD4$^+$CD25$^+$ T-cell population. The red box denotes the isotype control-defined gating threshold applied to discriminate specific Foxp3-positive staining from nonspecific background fluorescence and to establish the positive Treg gate. (B) Quantification of CD8$^+$ T cells expressed as a percentage of CD3$^+$ tumor-infiltrating lymphocytes (left) and the CD8$^+$/Treg ratio (right). Although no statistically significant difference in CD8$^+$ T cell frequency was observed between groups, a trend toward an increased CD8$^+$/Treg ratio was noted in the diphenidol-treated group.

A

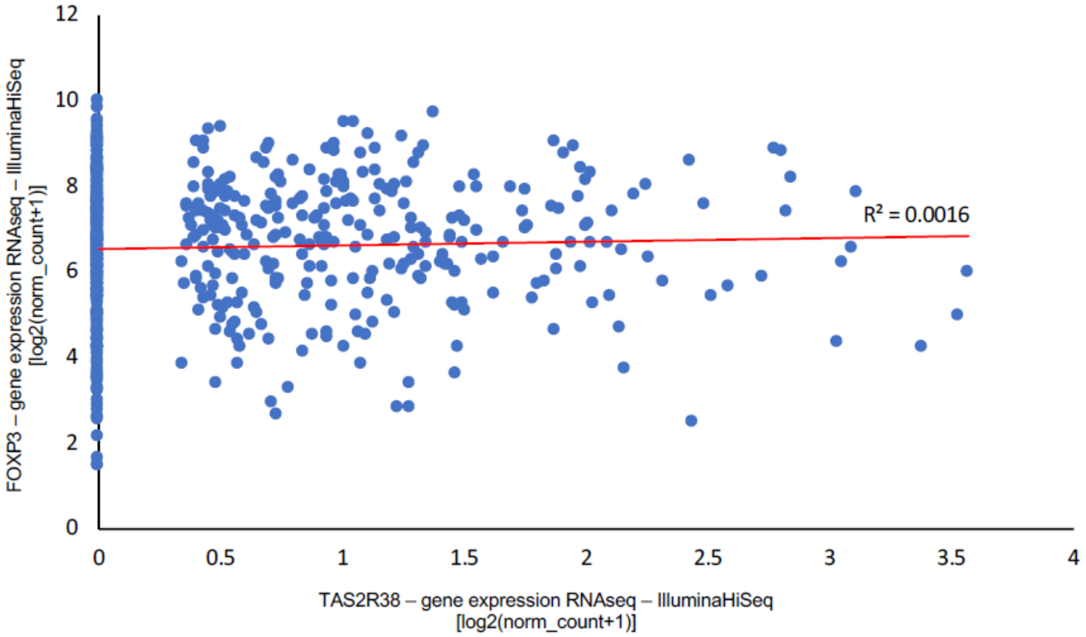

B

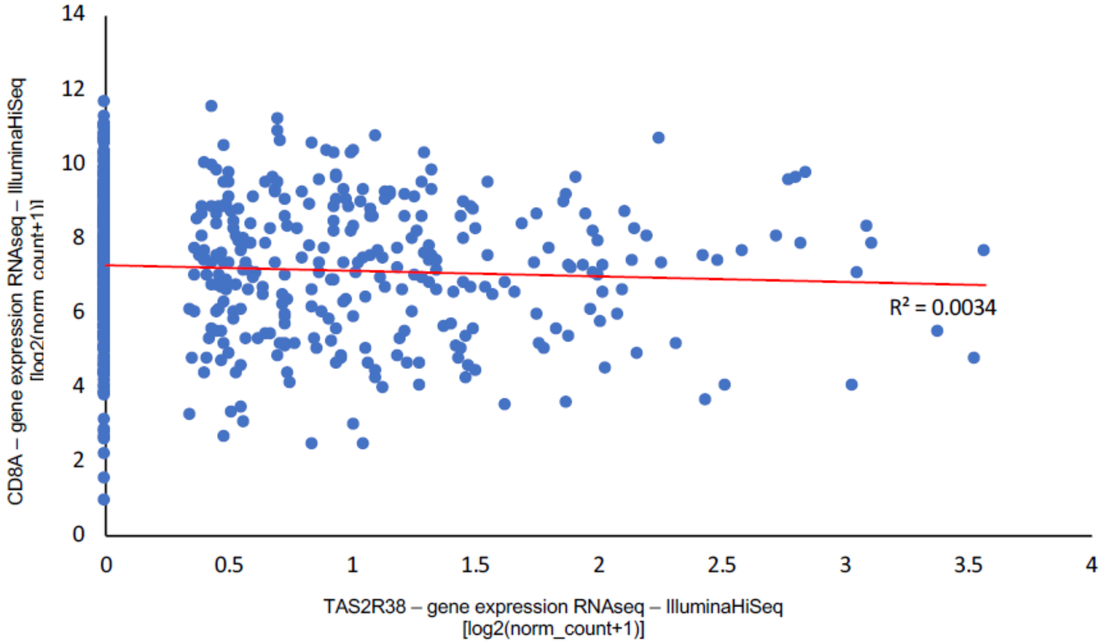

Figure S6. Correlation of TAS2R38 with immune-related markers in HNSC. (A) Scatter plot showing the correlation between TAS2R38 and FOXP3 expression in HNSC samples. (B) Correlation between TAS2R38 and CD8A expression in the same cohort.

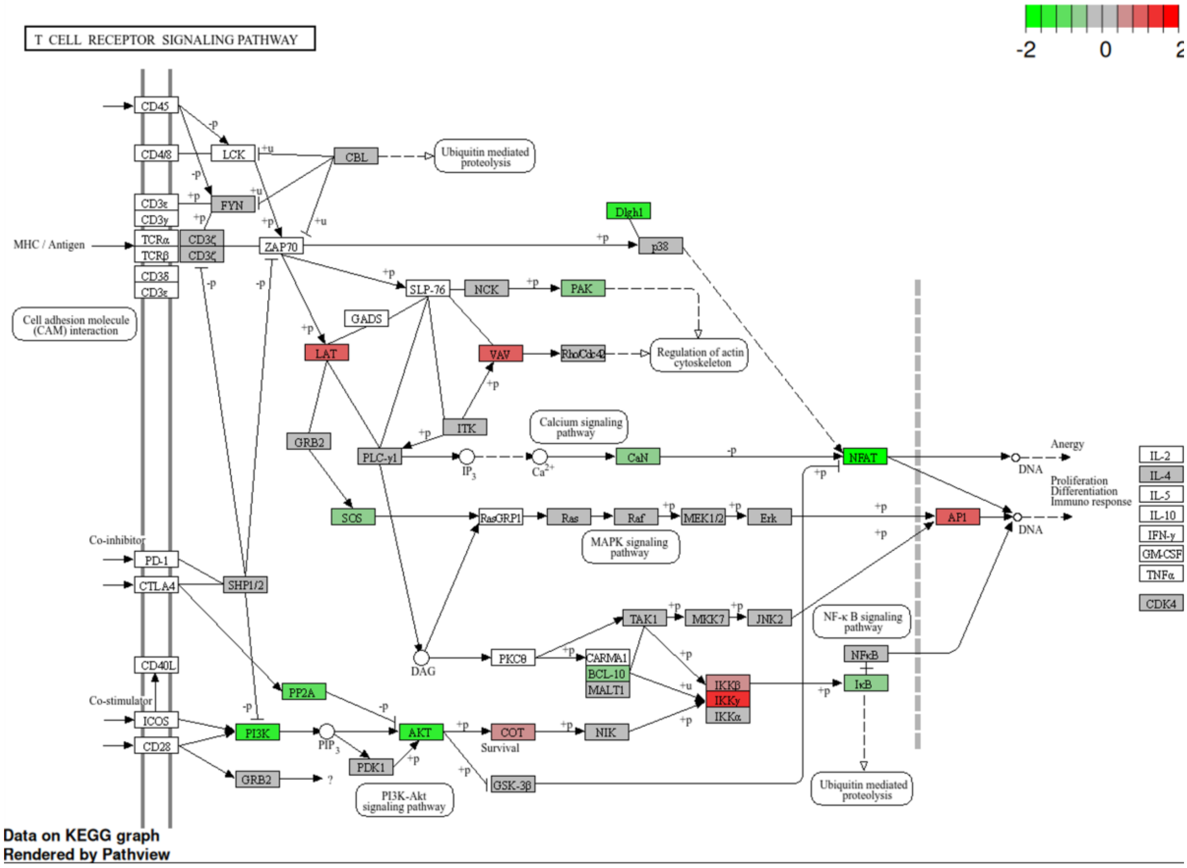

Figure S7. Mapping of differentially expressed genes onto the T cell receptor signaling pathway. Differentially expressed genes following TAS2R agonist treatment were mapped onto the T cell receptor signaling pathway using KEGG pathway visualization tools. Upregulated genes are indicated in red, whereas downregulated genes are shown in green. Although this pathway was not significantly enriched in KEGG analysis, several key signaling components, including NFAT, AP-1, AKT, and PI3K, exhibited altered expression, suggesting partial modulation of T cell–related signaling processes.

Table S1. Primer sequences of genes used for quantitative reverse transcription PCR.

Gene	Sequence (5'-3')
βactin-F	GGCTTTGCACATGCCGGA
βactin-R	TCTTTTGTGTCTTGATAGTTCGCC
Tas2r105-F	TGGACTGGGCCAAGAACAAT
Tas2r105-R	ATCCTGGAAGTTGCTAAGCCG
Tas2r137-F	ACATCAGACTGAAGCGACAGG
Tas2r137-R	CAGAAGGTAGCAACCAGGG
Il1rl1-F	CAAGTAGCCTCACGGCTCTG
Il1rl1-R	GCTCTCTGAGGTAGGGTCCA
Lzts2-F	AGTAGAGTTCTATCACGGCGAAG
Lzts2-R	GGGGCAACCCAGCGTCA
Heca-F	AGTCGGTTCTGCAGCCTATG
Heca-R	CACTAAAGTGGGCTGGGGAG
Sipa1l1-F	CAGTAAGGACCCCTCTCCCA
Sipa1l1-R	TCTGAGAGCATTTACCTTCTTCAA

F forward; R reverse.
